# A multicenter prospective randomized controlled trial investigating the effects of combustion-free nicotine alternatives on cardiovascular risk factors and metabolic parameters in individuals with type 2 diabetes who smoke: the DiaSmokeFree study protocol

**DOI:** 10.1007/s11739-023-03467-6

**Published:** 2023-11-24

**Authors:** Arkadiusz Krysiński, Cristina Russo, Davide Campagna, Antonino Di Pino, Sarah John, Jonathan Belsey, Pasquale Caponnetto, Lorina Vudu, Chong Wei Lim, Agostino Di Ciaula, Nicola Veronese, Mario Barbagallo, Farrukh Iqbal, David Fluck, Edward Franek, Riccardo Polosa, Pankaj Sharma, the DiaSmokeFree investigators

**Affiliations:** 1 Polish Academy of Sciences, Mossakowski Medical Research Centre Polish–Academy of Sciences, Warsaw, Poland; 2 Department of Internal Diseases, Endocrinology and Diabetology, Central Clinical Hospital, PIM MSWiA, Warsaw, Poland; 3 Ashford and Saint Peter’s Hospitals NHS Trust, Chertsey, UK; 4 Emergency Department, Teaching Hospital Policlinico “G. Rodolico–San Marco” of Catania, University of Catania, Catania, Italy; 5 Center of Excellence for the Acceleration of HArm Reduction (CoEHAR), University of Catania, Catania, Italy; 6 Department of Clinical and Experimental Medicine, University of Catania, Catania, Italy; 7 School of Clinical Medicine, University of Cambridge, Cambridge, UK; 8 JB Medical Ltd, Sudbury, UK; 9 Department of Educational Sciences, Section of Psychology, University of Catania, Catania, Italy; 10 Nicolae Testemitanu State Medical and Pharmaceutical University, Chisinau, Republic of Moldova; 11 Division of Internal Medicine, Department of Preventive and Regenerative Medicine and Ionian Area (DiMePrev-J), Clinica Medica “A. Murri”, University of Bari Aldo Moro, Bari, Italy; 12 Geriatrics Section, Department of Medicine, University of Palermo, Palermo, Italy; 13 The University of Lahore University College of Medicine and Dentistry, Lahore, Pakistan

**Keywords:** Diabetes, Smoking cessation, Tobacco harm reduction, e-Cigarettes, Heated tobacco products, Metabolic syndrome, Cardiovascular risk factors, Glycemic control

## Abstract

Stopping smoking is crucial for public health and especially for individuals with diabetes. Combustion-free nicotine alternatives like e-cigarettes and heated tobacco products are increasingly being used as substitutes for conventional cigarettes, contributing to the decline in smoking prevalence. However, there is limited information about the long-term health impact of those products in patients with diabetes. This randomized controlled trial aims to investigate whether switching from conventional cigarettes to combustion-free nicotine alternatives will lead to a measurable improvement in cardiovascular risk factors and metabolic parameters over a period of 2 years in smokers with type 2 diabetes. The multicenter study will be conducted in seven sites across four countries. A total of 576 smokers with type 2 diabetes will be randomly assigned (1:2 ratio) to either standard of care with brief cessation advice (Control Arm) or combustion-free nicotine alternatives use (Intervention Arm). The primary end point is the change in the proportion of patients with metabolic syndrome between baseline and the 2-year follow-up. Additionally, the study will analyze the absolute change in the sum of the individual factors of metabolic syndrome at each study time point. Patient recruitment has started in September 2021 and enrollment is expected to be completed by December 2023. Results will be reported in 2026. This study may provide valuable insights into cardiovascular and metabolic health benefits or risks associated with using combustion-free nicotine alternatives for individuals with type 2 diabetes who are seeking alternatives to tobacco cigarette smoking. The study protocol, informed consent forms, and relevant documents were approved by seven ethical review boards. Study results will be disseminated through articles published in high-quality, peer-reviewed journals and presentations at conferences.

## Introduction

Diabetes is a major public health problem and its burden is increasing globally [1]. People with diabetes have increased health risks mainly secondary to the development of macrovascular (coronary artery disease, stroke, and peripheral arterial disease) and microvascular complications (nephropathy, retinopathy, and diabetic neuropathy) [2]. Cardiovascular complications are the most common, often being the cause of death [3].

In addition to diabetes and hyperglycemia, well-established cardiovascular risk factors encompass obesity, hypertension, dyslipidemia, and other factors falling under the umbrella of metabolic syndrome (MetS). Lifestyle plays a critical role in the determination of cardiovascular risk in people with diabetes, independently from factors reflecting MetS and diabetes severity. In this context, the coexistence of cigarette smoking is of particular relevance.

People with diabetes who smoke cigarettes are at increased risk of cardiovascular events and premature death and avoiding smoking can reduce this risk [3–5]. Given that exposure to cigarette smoke is associated with vascular damage, endothelial dysfunction, and activation of coagulation and fibrinolysis [6–8], it is not surprising that smoking exacerbates the detrimental effects of elevated blood glucose and other risk factors, accelerating vascular damage in people with diabetes.

While reducing exposure to cigarette smoke is important for public health, it becomes even more significant for people with Type 2 diabetes mellitus (T2D). Prevalence of cigarette consumption has significantly decreased, but this trend has not been observed in those with diabetes [9–11], justifying an urgent need to target this vulnerable population with effective smoking cessation interventions, including counseling and nicotine-containing preparations [5, 12–15].

Despite high-quality evidence supporting the efficacy of combining smoking cessation medications with counseling [12–15], the evidence for efficacious smoking cessation interventions in people with diabetes is limited [5, 16, 17]. Consequently, novel, and more efficient approaches are required.

Although not authorized as medications for smoking cessation, combustion-free technologies for nicotine delivery, such as e-cigarettes (ECs) and heated tobacco products (HTPs), have become de facto harm reduction tools from cigarette smoke [18–20] and aids for quitting smoking [21, 22]. Currently, there is insufficient evidence to recommend use of ECs or HTPs as a tobacco cigarette substitute among people with diabetes who smoke, and there are no long-term studies assessing cardiovascular risk or the impact on metabolic parameters in people with diabetes who use these technologies.

The DiaSmokeFree investigators aim to determine whether smokers with T2D who switch to combustion-free nicotine alternatives (C-F NA) experience measurable improvements in their cardiovascular risk factors and metabolic parameters. The protocol of this prospective, randomized, controlled trial is designed to provide valuable insights into the potential benefits or risks associated with using C-F NA for individuals with T2D who are seeking alternatives to tobacco cigarette smoking, particularly in relation to their cardiovascular and metabolic health.

## Methods

The study is designed to assess the impact of combustion-free nicotine alternatives (C-F NA) on cardiovascular risk factors and metabolic homeostasis as a consequence of avoiding individual exposure to cigarette smoke toxicants in smokers with T2D. It is a 24-month prospective international, multicenter, open-label, randomized, controlled, two parallel-arm study conducted in seven locations across four different countries.

## Study population

Participants will be recruited on a voluntary basis from a group of cigarette smokers who have been clinically diagnosed with T2D. Only patients who smoked daily for at least 5 years will be considered for inclusion in the study. To confirm the smoking status of potential participants, an exhaled carbon monoxide (eCO) measurement will be taken at the screening visit. The cutoff for confirmation as a smoker will be an exhaled CO level greater than or equal to 7 parts per million (ppm). To avoid including patients with poorly controlled diabetes requiring immediate modification of therapy, it was decided to set a glycated hemoglobin percentage of between 6 and 12% as an inclusion criterion. Another criterion is a BMI between 17.6 kg/m^2^ and 34.5 kg/m^2^ to achieve greater homogeneity of the group and to avoid subjects with morbid obesity and coexisting other metabolic disorders, which could affect the outcome of the study. Each participant identified as a smoker with T2D will be offered access to free smoking cessation programs aimed at helping participants quit smoking. Only those who refuse to participate in the smoking cessation programs and express willingness to switch to C-F NA will be eligible for randomization. Before randomization, participants will provide informed consent.

Participants will be required to satisfy all of the following criteria.

Inclusion criteria:

- Men or women diagnosed with T2D (as defined by the American Diabetes Association).
- Cigarette smokers of ≥ 10 cigarettes/day (max 30 cigarettes/day).
- History of regular smoking for at least 5 consecutive years.
- Verified smoking status (eCO ≥ 7 ppm).
- Willingness to switch to a C-F NA.
- Refusal to participate in smoking cessation programs.
- Glycated hemoglobin between 6 and 12%.
- Body mass index between 17.6 kg/m^2^ and 34.5 kg/m^2^, both inclusive.
- Body weight exceeding at least 50 kg (men) or 40 kg (women).

Exclusion criteria:

- History of recent (i.e., within 4 weeks prior to visit 1) acute decompensation of T2D requiring change of treatment.
- Known clinically significant cardiovascular, metabolic, respiratory, renal, psychiatric, or other major disorder that, in the opinion of the principal investigator, would jeopardize the safety of the participant or impact on the validity of the study results.
- Any other condition or therapy that would make the patient unsuitable for the studies and will not allow participation for the full planned study period (e.g., active malignancy or other condition limiting life expectancy to < 12 months).
- Desire to quit smoking within the next 6 months.
- A significant history of alcohol or drug abuse within 24 months prior to screening.
- Regular use of any nicotine (e.g., e-cigarettes, NRT, nicotine pouches) or tobacco product (e.g., heated tobacco products—HTPs, oral smokeless) other than their own cigarettes within 14 days of screening.
- Pregnant or breast-feeding or intention to become pregnant during the study.
- Active participation in another clinical trial.

### Study design

**Fig. 1:**
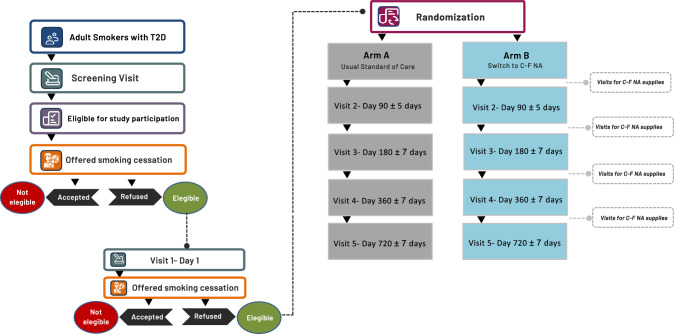
Study design flow of DiaSmokeFree; *V* visit, *C-F NA* combustion-free nicotine alternatives

The study follows a randomized controlled design with two parallel groups: control (A) and intervention (B) (Fig. 1). The study will be conducted in seven locations across four different countries (UK, Italy, Poland, and Moldova). The setting for the study will be an ambulatory (outpatient) setting. The design of the trial follows the rules set by the Standard Protocol Items: Recommendations for Interventional Trials (SPIRIT) guidelines (SPIRIT checklist).

Participants will attend a screening visit within 28 days prior to study visit 1 (Tables 1 and 2). During the screening visit, socio-demographic data, detailed medical history (including medication use), and detailed history of smoking, vaping, and heated tobacco products (HTPs) use will be collected. Additionally, participants’ intention to quit smoking will be assessed. All patients will be offered a smoking cessation program according to the local guidelines. A second opportunity to enroll in a free smoking cessation program will be offered before study enrollment. Any participant who expresses the intention of booking for the cessation program or to quit smoking in the next 6 months will be urged to do so and not be recruited in the study. Patients taking part in the study will be informed that they are free to quit smoking and withdraw from the study at any time. Moreover, participants will be encouraged to quit smoking at every contact time point throughout the whole study.

**Table 1:** Study assessments (Control group—Arm A)

Procedure	Screen (V0)	Baseline (V1) day 1	Week 12 (V2) day 90 ± 5	Week 24 (V3) day 180 ± 7	Week 52 (V4) day 360 ± 7	Week1 04 (V5) day 720 ± 7
Eligibility criteria check	X	X				
Medical Hx	X					
Medication use		X	X	X	X	X
Smoking/vaping Hx	X					
Cigarette consumption	X	X	X	X	X	X
Exhaled CO		X	X	X	X	X
FTND		X				
Intention to quit	X	X				
Informed consent		X				
Randomization		X				
Tracker APP installation		X				
Vital signs		X	X	X	X	X
Weight, BMI, waist circumference		X	X	X	X	X
Body composition equation*		X	X	X	X	X
QoL questionnaire		X	X	X	X	X
Smoking cessation advice	X	X	X	X	X	X
Spirometry		X				X
Metabolic syndrome assessment**		X	X	X	X	X
Blood sample***		X	X	X	X	X
Testosterone (men only)		X	X	X	X	X
Urine albumin to creatinine ratio (ACR)						
Safety reporting		X	X	X	X	X

*BL* baseline, *V* visit, *Hx* history, *CO* carbon monoxide, *BMI* body mass index, *FTND* Fagerstrom Test for Nicotine Dependence, *QoL* quality of life, *CBC* complete blood count, *WBC* white blood cells, *Hb* hemoglobin, *LDL* low-density lipoprotein, *HDL* high-density lipoprotein, *HbA1*_*C*_ glycated hemoglobin, *AE/SAE* (serious) adverse incidents

*Body composition (fat and skeletal muscle mass) by validated equation by Lean ME et al. (1996) and Al-Gindan YY et al. (2014)

**Metabolic syndrome is defined according to the National Cholesterol Education Program (NCEP) criteria as having ≥ 3 of the following five features:

1—fasting plasma glucose ≥ 6.1 mmol/L (110 mg/dL)

2—blood pressure ≥ 130/ ≥ 85 mmHg or treatment for hypertension

3—triglycerides ≥ 1.69 mmol/L (150 mg/dL) or treatment for high triglycerides

4—high-density lipoprotein (HDL) cholesterol < 1.29 mmol/L (50 mg/dL) for females or < 1.04 mmol/L (40 mg/dL) for males or treatment for low HDL

5—waist circumference ≥ 88 cm (35 in) for females or ≥ 102 cm (40 in) for males

***To measure: CBC (WBC Hb, platelets), lipid profile (triglycerides, LDL and HDL cholesterol) HbA1_c_, insulin level

**Table 2:** Study assessments (intervention group—Arm B)

Procedure	Screen (V0)	BL (V1)	Wk2	Wk4	Wk8	Wk12 (V2)	Wk20	Wk24 (V3)	Wk32	Wk40	Wk48	Wk52 (V4)	Wk60	Wk68	Wk76	Wk84	Wk92	Wk100	Wk104 (V5)
Eligibility criteria check	X	X																	
Medical Hx	X																		
Medication use		X				X		X				X							X
Smoking/vaping Hx	X																		
Cigarette consumption	X	X	X	X	X	X	X	X	X	X	X	X	X	X	X	X	X	X	X
Exhaled CO		X	X	X	X	X	X	X	X	X	X	X	X	X	X	X	X	X	X
FTND		X																	
C-F NA use			X	X	X	X	X	X	X	X	X	X	X	X	X	X	X	X	X
Intention to quit	X	X																	
Informed consent		X																	
Randomization		X																	
Tracker APP installation		X																	
C-F NA familiarization		X																	
Vital signs		X				X		X				X							X
Weight, BMI, waist circumference		X				X		X				X							X
Body composition equation*		X				X		X				X							X
QoL questionnaire		X				X		X				X							X
Smoking cessation advice	X	X	X	X	X	X	X	X	X	X	X	X	X	X	X	X	X	X	X
Spirometry		X																	X
Metabolic syndrome assessment**		X				X		X				X							X
Blood samples***		X				X		X				X							X
Urine albumin to creatinine ratio (ACR)		X				X		X				X							X
Testosterone (men only)		X				X		X				X							X
Safety reporting		X				X		X				X							X
Provide C-F NA device (+ 2 wks supply of consumables)		X																	
Hand out 2 wks supply of consumables			X																
Hand out 4 wks supply of consumables				X	X		X				X							X	
Hand out 2 × 4 wks supply of consumables						X		X	X	X		X	X	X	X	X	X		
Product use checks (collect used and unused consumables)			X	X	X	X	X	X	X	X	X	X	X	X	X	X	X	X	X

*BL* baseline, *V* visit, Hx history, *CO* carbon monoxide, *BMI* body mass index, *FTND* Fagerstrom Test for Nicotine Dependence, *QoL* quality of life, *CBC* complete blood count, *WBC* white blood cells, *Hb* hemoglobin, *LDL* low-density lipoprotein, *HDL* high-density lipoprotein, *HbA1*_*C*_ glycated hemoglobin, *AE/SAE* (serious) adverse incidents, *C-F NA* combustion-free nicotine alternatives

*Body composition (fat and skeletal muscle mass) by validated equation by Lean ME et al. (1996) and Al-Gindan YY et al. (2014)

**Metabolic syndrome is defined according to the National Cholesterol Education Program (NCEP) criteria as having ≥ 3 of the following five features:

1—fasting plasma glucose ≥ 6.1 mmol/L (110 mg/dL)

2—blood pressure ≥ 130/ ≥ 85 mmHg or treatment for hypertension

3—triglycerides ≥ 1.69 mmol/L (150 mg/dL) or treatment for high triglycerides

4—high-density lipoprotein (HDL) cholesterol < 1.29 mmol/L (50 mg/dL) for females or < 1.04 mmol/L (40 mg/dL) for males or treatment for low HDL

5—waist circumference ≥ 88 cm (35 in) for females or ≥ 102 cm (40 in) for males

***To measure: CBC (WBC Hb, platelets), lipid profile (Triglycerides, LDL and HDL cholesterol) HbA1_c_, insulin level

On day 1, baseline assessments will be performed for all participants (Tables 1 and 2). This includes measurements and evaluations related to cardiovascular risk factors, metabolic homeostasis, and general health. Following the baseline assessments, participants will be randomly assigned to either the control (A) arm or the intervention (B) arm. The randomization sequence will be computer generated, with an allocation ratio of 1:2 (arm A:arm B) to account for the estimated 50% proportion of subjects achieving sustained reduction in cigarette consumption of at least 80% in the arm B (see “Statistical Considerations”).

Participants randomized into the intervention arm (B) will be allowed to choose a preferred C-F NA (e-cigarettes, HTPs, etc.) from a given pool of popular options present in the respective markets. They will receive training and counseling on how to use the chosen device and will be provided with a full 2-week supply of the required consumables (cartridges/pods or e-liquids refill bottles for e-cigarettes, tobacco sticks for HTPs) on day 1. Participants randomized into the control arm (A) will be offered the usual standard of care, which includes brief smoking cessation counseling at each visit.

After randomization, participants’ smartphones will be equipped with a dedicated tracker application. This application is designed to monitor individual behaviors such as physical activity, adherence to capillary blood glucose monitoring, cigarette smoking frequency and daily C-F NA usage. The tracker application will also identify any protocol violations, collect adverse events and send reminders (e.g., next scheduled appointment, study restrictions, instructions) throughout the study duration. The use of a dedicated tracker application adds an innovative element to continuously collect data and enhances adherence to the study protocol.

After the baseline assessments (Visit 1), participants will be invited to attend four more clinical visits (Visits 2–5), which will be conducted in an ambulatory (outpatient) setting. During these visits, a number of measurements will be taken, with a particular focus on metabolic syndrome parameters, i.e., waist circumference, systolic blood pressure value, venous blood concentration of triglycerides, HDL, and fasting blood glucose (Tables 1 and 2). Also, two questionnaires will be used.

(Diabetes Quality of Life questionnaire and the Fagerström Test for Nicotine Dependence) to assess specific aspects of participants' health and smoking behavior.

Following each clinical visit, participants will be provided with an appropriate number of consumables (cartridges/pods or e-liquids refill bottles for e-cigarettes, tobacco sticks for HTPs) if they are in the intervention arm (Arm B). Participants in the intervention arm will have additional non-clinical visits between the clinical visits. These non-clinical visits are designed to replace any used consumables and provide an opportunity for study investigators to stimulate retention and check compliance. At these non-clinical visits, participants in Arm B will be required to return all empty, part-used, and unused consumables from the previous study period. This evaluation of the habitual pattern of C-F NA use and verification of product adherence are essential for monitoring the participants' compliance with the intervention.

Before each study visit, participants will be required to fast overnight (from midnight). During the clinic visits, clinical laboratory evaluations will be performed on fasting blood samples. Participants will also receive instructions to refrain from consuming alcohol for 24 h prior to clinic visits and will be advised not to consume more than 14 units of alcohol per week throughout the entire study duration. This is to minimize any potential interference of alcohol consumption with study outcomes and to maintain consistency in data collection. Throughout the study, any modifications in participants' diet and/or anti-diabetic medication will be regularly recorded and monitored.

At each visit, all participants will be advised and encouraged to completely quit smoking. They will be explicitly informed about the risks associated with smoking and will be offered referral to free smoking cessation programs at every contact time point.

Premature withdrawal from the study may occur if a participant experiences a severe adverse event (SAE), sustains any uncorrectable protocol deviations during the study, exhibits uncooperative behavior or non-attendance, decides to stop their participation at any time, becomes pregnant, or decides to accept the investigators advice to completely stop smoking.

The DiaSmokeFree study is designed as an unblinded trial due to the nature of the intervention, where participants and trial staff cannot be blinded to the specific intervention being provided (either C-F NA or brief smoking cessation counseling).

To ensure the integrity and reliability of the study data, the source data and source documents will be managed according to Good Clinical Practice (GCP) guidelines. The trial will formally conclude on the date of the last visit of the last patient in the last country participating in the trial.

For data collection, each individual patient will be allocated an electronic case report form (eCRF). Anonymized data from each study visit will be entered directly onto the eCRF, which will then serve as a source document for the trial. This data entry process ensures that data is collected accurately and consistently at all study sites. To maintain data quality, the study will use standardized instruments, such as the Diabetes Quality of Life questionnaire and the Fagerström Test for Nicotine Dependence, to assess specific aspects of participants' health and smoking behavior.

To protect participants’ privacy and confidentiality, each participant will be assigned a unique study identification number (patient ID). Participants' personal data will not be linked to the research results, and only a limited number of members of the research team will have access to the decoding list that links participant IDs to their personal information. All information obtained during the study procedures, including participant data and personal details, will be treated as private and confidential, in accordance with ethical and privacy regulations.

Compliance will be checked by counting all empty, partially used and unused consumables returned at each visit (i.e., consumables record checks) and by reviewing any non-compliance recorded in the research diary or via the tracker app.

### Patient and public involvement

In the DiaSmokeFree study, patient and public involvement consisted of a focus group of smokers with T2D. The focus group feedback was used to shape and refine the trial design, ensuring that the study is more relevant and responsive to the needs and expectations of its target population. Moreover, the study protocol has been reviewed by the Research and Development (R&D) Committee of Ashford and St Peter’s Hospitals NHS Foundation Trust, which includes a patient representative, thus ensuring that the study is ethically sound, aligns with patient interests, and respects patient rights. By involving patients and the public in the study design and review process, the DiaSmokeFree study demonstrates a commitment to patient-centered research.

### Objectives and end points

Primary end points: The primary objective of the study is to assess the impact of sustained use of C-F NA on the proportion of patients with metabolic syndrome (MetS) among individuals with T2D. The MetS will be defined by the National Cholesterol Education Program (NCEP) MetS score [23], and the primary end point will be the change in the prevalence of an NCEP MetS score below the diagnostic threshold (< 3) between baseline and the 2-year follow-up. The comparison will be made between patients with T2D in both arms of the study.

Secondary end points: The study will also assess the change in MetS prevalence at 3 months, 6 months, and 1 year as secondary outcomes. All assessments at each time point will be performed in all participants in both arms of the study.

Considering the results of several lifestyle modification interventions, the absolute reduction in MetS prevalence after smoking cessation is expected to be no less than 15% [24–28].

Additionally, the study will analyze the absolute change in the sum of the individual factors of MetS, as defined by NCEP criteria, at each study time point (between and within study groups. Other secondary end points will include changes in each individual factor of MetS and changes in the variables at each study time point (between and within study groups).

### Statistical considerations

#### Powering and sample size calculation

The sample size calculation is based on assumptions that the expected reduction in MetS prevalence after smoking cessation is 15% [24–28], and the expected baseline prevalence of MetS in T2D is 70% [29–32]. Sample size was calculated on the basis of demonstration of superiority, assuming a normal distribution of the sample [33]. Significance level was set at 5% (*α* = 0.05), with a power of 80% (*β* = 0.20). Based on these assumptions, the minimum number of subjects with analyzable data required is 160 per treatment arm (N). Considering an estimated 50% proportion of subjects in the intervention arm achieving sustained reduction in cigarette consumption of at least 80% for the study duration [34–38], the final number of subjects required in the intervention arm (N2) is set at 320. Taking into consideration the expected 20% withdrawal rate in both arms over 2 years, the total number of subjects recruited in each treatment arm is increased by this amount. Thus, the total number of subjects for both arms is set at 576.

#### Statistical analyses

The primary end point for the statistical analysis will be the between-groups difference in the calculated prevalence of MetS after at least 24 months of follow-up. The full analysis set (FAS) will include all subjects randomized to the intervention arm who achieve a sustained reduction in cigarette consumption of at least 80% during the follow-up, combined with all subjects randomized into the usual standard of care control group. Two approaches to the primary analysis will be used: (a) unadjusted analysis, based on a direct comparison of the change in prevalence using a Z test, and (b) adjusted analysis, where potential confounders (including age, gender, smoking, and medications for hypertension, hyperlipidemia, or diabetes) will be analyzed to identify factors that may influence the primary outcome. A generalized linear model will be used to adjust for identified confounders. Any difference between the groups will be assessed for statistical significance at a two-sided alpha of 0.05.

### Data monitoring and study safety

A Data Monitoring and Safety Committee (DMC) will be established before the study begins. The committee will periodically review safety data and make recommendations on whether to continue, modify, or stop the study based on the observed results. The DMC will be independent from the study sponsor and competing interests to ensure unbiased evaluation.

A trial monitoring plan will be developed based on the trial risk assessment. It may include on-site monitoring and will be agreed upon by the trial steering committee, and principal investigators. The clinical research organization (Metanoic Health Ltd) will arrange an independent monitor, separate from investigators and the sponsor, to ensure compliance with trial protocols and policies, participant protection, and accurate data collection.

Adverse events (AEs) and serious adverse events (SAEs) will be recorded throughout the study. Participants will be interviewed at each visit to investigate signs or symptoms. Participants will also be encouraged to report AEs/SAEs at any time. The investigator will gather sufficient information to determine the outcome and causality of AEs/SAEs and promptly notify the competent authority if necessary. Follow-up of AEs/SAEs will be required after the study's discontinuation if they persist or have sequelae.

### Ethical considerations and results dissemination

The study will adhere to the principles of Good Clinical Practice and the Declaration of Helsinki. It has been reviewed and approved by seven ethics committees. Relevant documentation, such as the informed consent form and patient information sheet, has been translated where necessary. The informed consent material will be obtained by site investigators through relevant forms. The chief investigator will be responsible for deciding whether amendments to the protocol are substantial or non-substantial. Substantial amendments will be submitted to the research ethics committee for approval before implementation.

In the UK, all investigators and trial site staff will comply with the Data Protection Act 2018 regarding the collection, storage, processing, and disclosure of personal information. In other countries, equivalent local data protection regulations will be followed.

The trial steering committee (TSC) will have access to the full trial data set. Site investigator(s) will need formal approval from the TSC and sponsor for data access requests. Committee members are independent and have no conflicts of interest.

The intention is to disseminate the study results through articles in peer-reviewed journals and conference presentations. A summary of results will be available on the Ashford and St Peter’s Hospitals’ website for public access. The anonymized data will be available to researchers upon request, following approval from the established scientific committee.

## Results

Patient recruitment has started in September 2021 and enrolment is expected to be completed by December 2023. Results will be reported in 2026.

## Discussion

Smoking cessation is a priority for patients with T2D and the use of C-F NA has grown exponentially in the past decade. However, there is limited knowledge regarding the overall health impact of C-F NA like e-cigarettes and HTPs on individuals with T2D who smoke. The DiaSmokeFree study will be the first trial to address this knowledge gap. The study has been specifically designed to tests the hypothesis that avoiding exposure to cigarette smoke toxicants by substituting tobacco cigarettes for C-F NA may lead to measurable amelioration in cardiovascular risk factors and metabolic parameters, compared to individuals who continue smoking tobacco cigarettes.

Although it is generally desirable, and in particular for individuals with T2D who smoke to stop any form of tobacco consumption, devices that deliver nicotine without combustion (i.e., C-F NA) are de facto replacing tobacco cigarettes among smokers worldwide [39–44]. Compared with tobacco cigarettes, they offer substantial reduction in exposure to toxic chemical emissions [45–49]. For this reason, they are proposed for harm reduction from cigarette smoke and for smoking cessation [18, 21, 50, 51]. However, C-F NA are not risk free, and they might have adverse health effects not yet identified.

DiaSmokeFree's study design was based on the idea that C-F NA are marketed as substitutes for tobacco cigarettes and that the reference product would be participants’ own brand tobacco cigarettes. The study duration of 24 months allows for observation of changes in the primary end point, and the randomized design ensures high-quality data by equalizing variation in smoking history and other variables between the study arms. Additionally, the welfare of participants is emphasized throughout the study with regular reminders to quit smoking and access to free smoking cessation programs.

Several of the parameters measured in this study are associated with the development of cardiovascular diseases (e.g., hypertension, elevated blood cholesterol, and BMI > 25), and some of these parameters have been shown to improve relatively quickly after smoking cessation [34, 35]. Therefore, the profile of these changes after switching to C-F NA may provide valuable insight into the overall potential of these products to reduce the overall cardiovascular risk.

The introduction of several compliance checkpoints throughout the study to record the consumption of tobacco cigarettes and use of C-F NA is a significant feature of the study. Ensuring compliance with the study protocol is crucial for obtaining reliable results as failure to replace conventional cigarettes fully or largely with C-F NA would reduce or nullify the expected changes in study end points. Participants will be encouraged to adhere to their assigned intervention and reduce conventional cigarette consumption by at least 80% from baseline. They also will be informed that a compliance check will be performed at each visit through biochemical verification. Compliance will be also checked by counting all empty, partially used and unused consumables returned at each visit (i.e., consumables record checks) and by reviewing any non-compliance recorded in the research diary or via the tracker app.

Although compliance in this study is not expected to differ significantly compared to other similar studies, our power calculation is overestimated, to take account of a non-compliance rate of 50%. Therefore, the C-F NA population will be oversampled using a 1:2 randomization ratio scheme (i.e., for every patient randomized in the control population, two patients will be randomized in the C-F NA population). Finally, by requiring participants to return to the clinic to regularly stock up on cartridges/pods or e-liquids refill bottles for e-cigarettes, and tobacco sticks for HTPs will also increase study participation and retention in the C-F NA population.

The study’s innovative features include offering participants a range of C-F NA options to choose from, ensuring a user-friendly experience, and increasing the likelihood of adoption. This element of choice is missing from any switching trials to date, in which a single product is generally offered. Since DiaSmokeFree's population sample consists mainly of elderly patients, we will provide devices that ensure the most user-friendly experience possible (e.g., easy-to-refill consumables, pre-filled consumables, etc.). This approach enhances generalizability and makes the findings applicable to a broader population.

Another significant feature that makes this study unique is the incorporation of an innovative tracker application designed to monitor various behaviors and enhance adherence to the study protocol. The application serves multiple functions, including monitoring physical activity, adherence to capillary blood glucose monitoring, cigarette smoking frequency and daily C-F NA usage. By continuously collecting data through the tracker application, researchers can closely monitor participants' behaviors and identify any protocol violations. This real-time monitoring allows for prompt intervention or corrective measures to maintain adherence and ensure the integrity of the study. Additionally, the tracker application enables the collection of adverse events, which provides valuable information on the safety and tolerability of C-F NAs. Researchers can promptly address any adverse events and take necessary actions to safeguard participant well-being. Furthermore, the application serves as a communication tool, sending reminders to participants about their next scheduled appointment, study restrictions, and instructions. These reminders help to keep participants engaged and informed throughout the entire duration of the study.

As with any study, our protocol has limitations. First, subject retention in the study can be challenging due to the relatively long follow-up (24 months). Nonetheless, inviting participants to return to the clinic with study equipment provided free of charge and a dedicated fast-track approach to their outpatient appointments may increase study participation and retention. Second, the results of DiaSmokeFree cannot be extrapolated to all smokers with diabetes. We will not recruit smokers with untreated disease or with acute complications of T2D. Also, individuals with Type 1 Diabetes (T1D) will be excluded from the study. The impact of COVID-19 restrictions on recruitment is also acknowledged, and strategies will be implemented to mitigate any potential drawbacks.

## Conclusions

The evidence for potential risk/harm reduction with long-term use of C-F NA is virtually unexplored in patients with T2D. DiaSmokeFree's data will be an important addition to the growing body of knowledge about the health effects of C-F NA and will provide valuable progress into the overall potential of these products to reduce the cardio-metabolic risk, especially in people with diabetes. For governments, health authorities, and health professionals to be able to provide guidance on cigarette substitution, reliable evidence-based information about risk reduction and harm reversal in T2D is needed. The DiaSmokeFree RCT will stand out as the first clinical trial being adequately powered to collect such evidence.

## Data Availability

Direct access to the study data will be granted to authorised representatives from the Sponsor, host institution and the regulatory authorities to permit trial-related monitoring, audits and inspections- in line with participant consent. Requests to access data from other investigators will be assessed by a scientific committee chaired by Prof. Pankaj Sharma and composed of one member from each participating site as well as an independent statistical advisor. It will be the guiding principle of this scientific committee that no reasonable request for data will be refused.

## References

[1] GBD Diseases and Injuries Collaborators (2020). Global burden of 369 diseases and injuries in 204 countries and territories, 1990–2019: a systematic analysis for the Global Burden of Disease Study 2019. Lancet.

[2] Fowler MJ (2011). Microvascular and macrovascular complications of diabetes. Clin Diabetes.

[3] Pan A, Wang Y, Talaei M, Hu FB (2015). Relation of smoking with total mortality and cardiovascular events among patients with diabetes mellitus: a meta-analysis and systematic review. Circulation.

[4] Qin R, Chen T, Lou Q (2013). Excess risk of mortality and cardiovascular events associated with smoking among patients with diabetes: meta-analysis of observational prospective studies. Int J Cardiol.

[5] Campagna D, Alamo A, Di Pino A, Russo C, Calogero AE, Purrello F, Polosa R (2019). Smoking and diabetes: dangerous liaisons and confusing relationships. Diabetol Metab Syndr.

[6] Cacciola RR, Guarino F, Polosa R (2007). Relevance of endothelial-haemostatic dysfunction in cigarette smoking. Curr Med Chem.

[7] Guarino F, Cantarella G, Caruso M, Russo C, Mancuso S, Arcidiacono G, Cacciola RR, Bernardini R, Polosa R (2011). Endothelial activation and injury by cigarette smoke exposure. J Biol Regul Homeost Agents.

[8] Caponnetto P, Russo C, Di Maria A, Morjaria JB, Barton S, Guarino F, Basile E, Proiti M, Bertino G, Cacciola RR, Polosa R (2011). Circulating endothelial-coagulative activation markers after smoking cessation: a 12-month observational study. Eur J Clin Invest.

[9] Stanton CA, Keith DR, Gaalema DE (2016). Trends in tobacco use among US adults with chronic health conditions: national survey on drug use and health 2005–2013. Prev Med.

[10] Ford ES, Mokdad AH, Gregg EW (2004). Trends in cigarette smoking among US adults with diabetes: findings from the behavioral risk factor surveillance system. Prev Med.

[11] Fan AZ, Rock V, Zhang X, Li Y, Elam-Evans L, Balluz L (2013). Trends in cigarette smoking rates and quit attempts among adults with and without diagnosed diabetes, United States, 2001–2010. Prev Chronic Dis.

[12] Polosa R, Benowitz NL (2011). Treatment of nicotine addiction: present therapeutic options and pipeline developments. Trends Pharmacol Sci.

[13] Stead LF, Koilpillai P, Fanshawe TR, Lancaster T (2016). Combined pharmacotherapy and behavioural interventions for smoking cessation. Cochrane Database Sys Rev.

[14] United States public health service office of the surgeon general, national center for chronic disease prevention and health promotion (US) office on smoking and health. Smoking cessation: a report of the Surgeon General. US department of health and human services; 2020. https://www.ncbi.nlm.nih.gov/books/NBK555591/. Accessed 8 May 2023

[15] Rigotti NA, Kruse GR, Livingstone-Banks J, Hartmann-Boyce J (2022). Treatment of tobacco smoking: a review. JAMA.

[16] Nagrebetsky A, Brettell R, Roberts N, Farmer A (2014). Smoking cessation in adults with diabetes: a systematic review and meta-analysis of data from randomised controlled trials. BMJ Open.

[17] Russo C, Walicka M, Caponnetto P, Cibella F, Maglia M, Alamo A, Campagna D, Frittitta L, Di Mauro M, Caci G, Krysinski A, Franek E, Polosa R (2022). Efficacy and safety of varenicline for smoking cessation in patients with type 2 diabetes: a randomized clinical trial. JAMA Netw Open.

[18] O’Leary R, Polosa R (2020). Tobacco harm reduction in the 21st century. Drugs Alcohol Today.

[19] Caruso M, Emma R, Distefano A, Rust S, Poulas K, Giordano A, Volarevic V, Mesiakaris K, Boffo S, Arsenijevic A, Karanasios G, Pulvirenti R, Ilic A, Canciello A, Zuccarello P, Ferrante M, Polosa R, Li VG (2022). Comparative assessment of electronic nicotine delivery systems aerosol and cigarette smoke on endothelial cell migration: the replica project. Drug Test Anal.

[20] Morjaria JB, Campagna D, Caci G, O’Leary R, Polosa R (2022). Health impact of e-cigarettes and heated tobacco products in chronic obstructive pulmonary disease: current and emerging evidence. Expert Rev Respir Med.

[21] Hartmann-Boyce J, Lindson N, Butler AR, McRobbie H, Bullen C, Begh R, Theodoulou A, Notley C, Rigotti NA, Turner T, Fanshawe TR, Hajek P (2022). Electronic cigarettes for smoking cessation. Cochrane Database Syst Rev.

[22] Caponnetto P, Campagna D, Maglia M, Benfatto F, Emma R, Caruso M, Caci G, Busà B, Pennisi A, Ceracchi M, Migliore M, Signorelli M (2023). Comparing the effectiveness, tolerability, and acceptability of heated tobacco products and refillable electronic cigarettes for cigarette substitution (CEASEFIRE): randomized controlled trial. JMIR Public Health Surveill.

[23] National Institute of Health (2001). Executive summary of the third report of the national cholesterol education program (NCEP) expert panel on detection, evaluation and treatment of high blood cholesterol in adults (Adult treatment panel III). JAMA.

[24] Didangelos TP, Thanopoulou AK, Bousboulas SH (2004). The ORLIstat and CArdiovascular risk profile in patients with metabolic syndrome and type 2 DIAbetes (ORLICARDIA) study. Curr Med Res Opin.

[25] Bo S, Ciccone G, Baldi C (2007). Effectiveness of a lifestyle intervention on metabolic syndrome. A randomized controlled trial. J Gen Intern Med.

[26] Christensen P, Bliddal H, Riecke BF (2011). Comparison of a low-energy diet and a very low-energy diet in sedentary obese individuals: a pragmatic randomized controlled trial. Clin Obes.

[27] Yoon NH, Yoo S, Kim H (2015). Routine screening and consultation facilitate improvement of metabolic syndrome. J Korean Med Sci.

[28] Sharip A, Firek A, Tonstad S (2017). The effects of smoking cessation on the risk factors for the metabolic syndrome: a follow-up study of veterans. J Smok Cessat.

[29] Bonadonna RC, Cucinotta D, Fedele D (2006). The metabolic syndrome is a risk indicator of microvascular and macrovascular complications in diabetes: results from Metascreen, a multicenter diabetes clinic-based survey. Diabetes Care.

[30] Lin SX, Pi-Sunyer EX (2007). Prevalence of the metabolic syndrome among US middle-aged and older adults with and without diabetes: a preliminary analysis of the NHANES 1999–2002 data. Ethn Dis.

[31] Yadav D (2013). Prevalence of metabolic syndrome in type 2 diabetes mellitus using NCEP-ATPIII, IDF and WHO definition and its agreement in gwalior chambal region of central India. Glob J Health Sci.

[32] Song SH, Hardisty CA (2008). Diagnosing metabolic syndrome in type 2 diabetes: does it matter?. QJM.

[33] Pocock SJ (1983). Clinical trials: a practical approach.

[34] Polosa R, Morjaria JB, Caponnetto P (2016). Blood pressure control in smokers with arterial hypertension who switched to electronic cigarettes. Int J Environ Res Public Health.

[35] Polosa R, Morjaria JB, Caponnetto P (2016). Persisting long term benefits of smoking abstinence and reduction in asthmatic smokers who have switched to electronic cigarettes. Discov Med.

[36] Polosa R, Morjaria JB, Prosperini U (2018). Health effects in COPD smokers who switch to electronic cigarettes: a retrospective-prospective 3-year follow-up. Int J Chron Obstruct Pulmon Dis.

[37] Adriaens K, Van Gucht D, Declerck P (2014). Effectiveness of the electronic cigarette: an eight-week flemish study with six-month follow-up on smoking reduction, craving and experienced benefits and complaints. Int J Environ Res Public Health.

[38] Polosa R, Caponnetto P, Maglia M (2014). Success rates with nicotine personal vaporizers: a prospective 6-month pilot study of smokers not intending to quit. BMC Public Health.

[39] European Commission 2021 Special Eurobarometer 506: attitudes of Europeans towards tobacco and electronic cigarettes. European Commission. https://data.europa.eu/data/datasets/s2240_506_eng?locale=en. Accessed 16 May 2023

[40] McNeill A, Brose L, Calder R, Simonavicius E, Robson D 2021 Vaping in England: evidence update February 2021: a report commissioned by Public Health England. London: public health England. https://www.gov.uk/government/publications/vaping-in-england-evidence-update-february-2021. Accessed 29 Apr 2023

[41] Jerzyński T, Stimson GV, Shapiro H, Król G (2021). Estimation of the global number of e-cigarette users in 2020. Harm Reduct J.

[42] Hori A, Tabuchi T, Kunugita N (2020). Rapid increase in heated tobacco product (HTP) use from 2015 to 2019: from the Japan ‘society and new tobacco’ internet survey (JASTIS). Tob Control.

[43] Ratajczak A, Jankowski P, Strus P, Feleszko W (2020). Heat not burn tobacco product-a new global trend: impact of heat-not-burn tobacco products on public health, a systematic review. Int J Environ Res Public Health.

[44] Miller CR, Sutanto E, Smith DM, Hitchman SC, Gravely S, Yong HH, Borland R, O'Connor RJ, Cummings KM, Fong GT, Hyland A, Quah ACK, Goniewicz ML (2022). Characterizing heated tobacco product use among adult cigarette smokers and nicotine vaping product users in the 2018 ITC four country smoking and vaping survey. Nicotine Tob Res.

[45] Haziza C, de La Bourdonnaye G, Merlet S, Benzimra M, Ancerewicz J, Donelli A, Baker G, Picavet P, Lüdicke F (2016). Assessment of the reduction in levels of exposure to harmful and potentially harmful constituents in Japanese subjects using a novel tobacco heating system compared with conventional cigarettes and smoking abstinence: a randomized controlled study in confinement. Regul Toxicol Pharmacol.

[46] Gale N, McEwan M, Camacho OM, Hardie G, Murphy J, Proctor CJ (2021). Changes in biomarkers of exposure on switching from a conventional cigarette to the glo tobacco heating product: a randomized. Controll Ambul Study Nicotine Tob Res.

[47] Farsalinos KE, Polosa R (2014). Safety evaluation and risk assessment of electronic cigarettes as tobacco cigarette substitutes: a systematic review. Ther Adv Drug Saf.

[48] Daynard R (2018). Public health consequences of e-cigarettes: a consensus study report of the national academies of sciences, engineering, and medicine. J Public Health Pol.

[49] Polosa R, O'Leary R, Tashkin D, Emma R, Caruso M (2019). The effect of e-cigarette aerosol emissions on respiratory health: a narrative review. Expert Rev Respir Med.

[50] Polosa R, Rodu B, Caponnetto P, Maglia M, Raciti C (2013). A fresh look at tobacco harm reduction: the case for the electronic cigarette. Harm Reduct J.

[51] Caruso M, Emma R, Distefano A, Rust S, Poulas K, Zadjali F, Giordano A, Volarevic V, Mesiakaris K, Al Tobi M, Boffo S, Arsenijevic A, Zuccarello P, Giallongo C, Ferrante M, Polosa R, Li Volti G, Replica Project Group (2021). Electronic nicotine delivery systems exhibit reduced bronchial epithelial cells toxicity compared to cigarette: the Replica Project. Sci Rep.

